# Feasibility and Acceptability of Online Culturally Adapted Cognitive Behavioural Therapy for Depression and Anxiety in Canadians of South Asian Origin: A Randomized Controlled Trial: Faisabilité et acceptabilité de la thérapie cognitivo-comportementale en ligne adaptée à la culture pour traiter la dépression et l’anxiété chez les Canadiens d’origine sud-asiatique : Essai contrôlé à répartition aléatoire

**DOI:** 10.1177/07067437251337644

**Published:** 2025-05-15

**Authors:** Farooq Naeem, Muhammad Ishrat Husain, Muhammad Omair Husain, Baldev Mutta, Gary Thandi, Azaad Kassam, Maureen Abbott, Marcos Sanches, Helen-Maria Vasiliadis, Saeed Farooq, Kwame McKenzie

**Affiliations:** 1 Department of Psychiatry, 7978 University of Toronto & Center for Addiction and Mental Health, Toronto, Canada; 2 Punjabi Community Health Services, Toronto, Canada; 3 Moving Forward Family Services, Vancouver, Canada; 4 Queensway-Carleton Hospital, 6363 University of Ottawa, Ottawa, Canada; 5 Mental Health Commission of Canada, Ottawa, Canada; 6 Université de Sherbrooke, Sherbrooke, Quebec, Canada; 7 Institute for Global Health and Wellbeing, Keele University, Keele, England

**Keywords:** culture, cognitive, therapy, South, Asian, online

## Abstract

**Background:**

This paper reports a pilot trial of culturally adapted CBT (CaCBT) for Canadian South Asians. The primary objective of this study was to assess the feasibility and acceptability of online CaCBT to treat anxiety and depression in Canadian South Asian individuals. The secondary objective was to measure changes in depression, anxiety, and disability.

**Methods:**

An assessor-blind randomized clinical trial was conducted at 3 sites in Canada (Greater Toronto Area, Ottawa, and Vancouver). One hundred forty-six participants were randomly allocated to 1 of 2 groups: Ca-CBT (experimental group) or standard cognitive behavioural therapy (CBT) (control group) in a 1:1 ratio. The primary outcome, feasibility, was measured through engagement, recruitment, and participant retention. Acceptability was measured through the Verona Service Satisfaction Scale. Working Alliance Inventory was used to measure therapeutic engagement. Secondary outcomes included depression (Hospital Anxiety and Depression Scale—HADS), somatic symptoms (Bradford Somatic Inventory—BSI), and disability (WHO Disability Assessment Schedule 2.0 (WHODAS). Assessments were carried out at baseline, at the end of therapy (12 weeks from baseline), and at follow-up (36 weeks from baseline).

**Results:**

We were able to recruit participants within the given timeframe with excellent retention rates in both arms. Most participants in the intervention group, 56 (74.7%), attended ≥ 8 sessions, and 11 (14.7%) attended 5 to 7 sessions. Eight (10.7%) participants from the intervention group and 9 (12.0%) from the control group dropped out of therapy (<5 sessions). Participants in the intervention group reported higher levels of satisfaction (*P* = 0.001) and therapeutic engagement (*P* < 0.001) compared with the control group. Participants in both groups benefited from CBT.

**Conclusions:**

This is the first report of online CaCBT for depression and anxiety for Canadian South Asians. The intervention is acceptable and feasible. Culturally adapted CBT is as effective as standard CBT in reducing the symptoms of depression and anxiety.

## Abrégé

**Contexte:**

Le présent article fait le rapport d’un essai pilote de TCC adaptée à la culture (TCCaC) pour les Canadiens d’origine sud-asiatique. Le principal objectif de cette étude était d’évaluer la faisabilité et l’acceptabilité de la TCCaC en ligne pour traiter l’anxiété et la dépression chez les Canadiens d’origine sud-asiatique. L’objectif secondaire était de mesurer l’évolution de la dépression, de l’anxiété et de l’invalidité.

**Méthodes:**

Un projet de recherche à répartition aléatoire, mené à l’insu de l’évaluateur, a été mené à trois endroits au Canada (région du Grand Toronto, Ottawa et Vancouver). 146 participants ont été répartis au hasard, en proportions égales, dans l’un des deux groupes suivants : TCCaC (groupe expérimental) ou TCC standard (groupe témoin). Le résultat principal, la faisabilité, a été mesuré par l’engagement, le recrutement et la rétention des participants. L’acceptabilité a été mesurée au moyen de l’échelle Verona Service Satisfaction Scale. L’échelle Working Alliance Inventory (WAI) a été utilisée pour mesurer l’engagement thérapeutique. Les résultats secondaires comprenaient la dépression (Hospital Anxiety and Depression Scale – HAD), les symptômes somatiques (Bradford Somatic Inventory – BSI) et l’incapacité (Disability Assessment Schedule 2.0 de l’OMS – WHODAS). Les évaluations ont été effectuées au niveau initial, à la fin du traitement (12 semaines après la valeur initiale) et au moment du suivi (36 semaines après la valeur initiale).

**Résultats:**

Nous avons été en mesure de recruter des participants dans les délais impartis, avec d’excellents taux de rétention dans les deux groupes. La plupart des participants du groupe d’intervention (56, soit 74,7%) ont assisté à ≥ 8 séances et 11 (14,7%) à 5, 6 ou 7 séances. 8 participants (10,7%) du groupe d’intervention et 9 (12,0%) du groupe de contrôle ont abandonné le traitement (< 5 séances). Les participants du groupe d’intervention ont déclaré des niveaux plus élevés de satisfaction (*P* = 0,001) et d’engagement thérapeutique (*P* < 0,001) comparativement au groupe témoin. La TCC a été bénéfique pour les participants des deux groupes.

**Conclusions:**

Il s’agit du premier rapport sur la TCCaC en ligne pour traiter la dépression et l’anxiété chez les Canadiens d’origine sud-asiatique. L’intervention est acceptable et réalisable. La TCC adaptée à la culture est aussi efficace que la TCC standard pour réduire les symptômes de dépression et d’anxiété.

## Background

The Mental Health Commission of Canada (MHCC) recommends improving access to appropriate, effective, evidence-based treatments that meet the unique sociocultural needs of persons from a diverse background.^1^ Canadians of South Asian (SA) origin constitute 5.5% of the Canadian population, representing 25% of all Canadian minorities.^2^ SA Canadians are disproportionately impacted by the social determinants of health, including unemployment, low income, language barriers, low education, low literacy, and migration stress (Beiser & Edwards). The SA Canadians are affected by higher rates of mood and anxiety disorders, in particular, those immigrating to Canada at age 17 or younger.^3,4^

SA Canadians with a major depressive episode are 85% less likely to seek treatment than Canadians who have experienced the same illness but identified as white.^5^ They also reported the highest proportion of unmet mental health care needs (48%) and the highest percentage of perceived barriers to the availability of mental health care (33%) as compared to other minority groups.^6^ Lesser use of mental health services by SA Canadians highlights the inequities concerning access to appropriate care for these populations.^5–8^

Since CBT is underpinned by the Western cultural values, it requires cultural adaptation when working with persons from non-Western cultural backgrounds.^9–12^ Culturally adapted cognitive behavioural therapy (CaCBT) is an evidence-based practice which can increase access to mental health services and improve outcomes for immigrant, refugee, ethnocultural and racialized minorities.

In a recent literature review, we identified 18 adaptation frameworks to culturally adapt psychotherapy.^13^ Most frameworks were based on therapists’ personal experiences of working with ethnic minority clients, especially American blacks, Latin, and Chinese.^13^ None of the frameworks was the direct outcome of research that engaged stakeholders. The majority of adaptations were made in the dimensions of language, context, concepts, family, communication, content, cultural norms and practices, context and delivery, therapeutic alliance, and treatment goals.^14^ These frameworks describe issues related to the cultural adaptation of therapies in general. The only adaptation framework that was the outcome of a series of qualitative studies and focuses only on CBT has been used in 6 countries to culturally adapt CBT and has been tested in more than 20 Randomized Controlled Trials (RCTs)^15^ recommends 3 areas of adaptation: (a) awareness of culture and religion (e.g., understanding the cause and effect model of mental illness used by the population in focus—bio-psycho-socio-spiritual model, language, and terminology since the literal translations don’t work, communication styles, idioms of distress and personal boundaries, family and caregivers’ involvement, and use of religious and spiritual coping strategies); (b) assessment and engagement (e.g., self-awareness in therapists about their own belief system, understanding of common presenting complaints and concerns, assessment of acculturation and immigration status, racism and racial or other trauma, stigma, shame and guilt, barriers to seeking therapy and engagement with therapy, awareness of illness, its causes, and its treatment and the beliefs about illness, its causes, and its treatment); and (c) adjustment in therapy (e.g., culturally acceptable patient–therapist relationship taking into consideration attitude towards authority in a given culture, psycho-education and access to therapy, cultural variations in dysfunctional beliefs, acceptable therapy settings and style, adjustments or modifications are required in therapy settings, use of culturally favourable communication strategies such as stories or images, understanding barriers in therapy such as how to ensure homework assignments are completed, and adjustments in therapy techniques).

CBT can be delivered through online platforms such as Zoom and Webex and has been reported to be effective, economical^16^ and potentially overcome challenges of cost, physical access, and availability of therapists,^17^ thus improving access for hard-to-reach, stigmatized and marginalized populations.^18^

Adapting CBT for the growing SA population in Canada can enhance equitable access to effective, culturally appropriate interventions. We report the first study of online CaCBT for depression and anxiety among the South Asian populations versus standard CBT.

## Objectives

The primary objective of this study was to assess the feasibility and acceptability of online CaCBT to treat anxiety and depression in Canadian SA individuals. The secondary objective was to measure changes in depression, anxiety, and disability.

## Methods

### Study Design

An assessor-blind randomized clinical trial was conducted at 3 sites in Canada (Greater Toronto Area, Ottawa, and Vancouver). Participants were randomly allocated to 1 of 2 groups at each site: CaCBT (experimental group) or standard CBT (control group) in a 1:1 ratio. The experimental group received 8–12 weekly sessions of CaCBT, while the control group received 8–12 weekly sessions of standard CBT. The study received ethics approval from the Centre for Addiction and Mental Health, Toronto, Canada (#071/2019). The study was conducted online due to the COVID pandemic.

### Participants

Depressed or anxious SA participants were recruited through a social media campaign and from participating agencies. Participants between the ages of 18 and 64 who scored >8 on the Hospital Anxiety and Depression Scale (HADS)—depression subscale or anxiety subscale were included in the study. Participants who used alcohol or drugs excessively and had significant cognitive impairment (e.g., intellectual disability or dementia) or active psychosis using DSM-5 criteria were excluded. Similarly, participants who had received CBT during the previous 12 months were also excluded.

### Trial Intervention-Online CaCBT

CBT was adapted through qualitative work guided by the Southampton adaption framework.^15,19^ The findings of this qualitative study have been described in detail elsewhere.^20^ In brief, to understand their perception of depression and anxiety and its treatment, individuals from 4 different target groups were recruited through our partnering agencies within the target local community: (a) SA individuals with depression and/or anxiety (*n* = 13); (b) caregivers and family members of SA individuals affected by depression and anxiety (*n* = 9); (c) mental health professionals and (*n* = 10); and (d) SA community opinion leaders (*n* = 10). Interviews and focus groups were conducted using topic guides used previously to adapt CBT.^21–27^ Information from these qualitative studies was used to culturally adapt an existing standard CBT for depression and anxiety manual^.28^ The adaptation process involved 2 consensus meetings with the Research Team and Expert Advisory Committee, and a detailed review was conducted to verify its accuracy. The CaCBT manual can be accessed at (https://www.camh.ca). Participants received 8–12 intervention sessions delivered online using Webex individually. The intervention was offered in English, and there was no therapist–participant matching since the idea behind CaCBT is to help therapists from 1 culture engage with persons from another.

### Control Group—Standard CBT

The participants in the control group received the same intervention, except that it was not culturally adapted. They received 8–12 sessions of standard CBT.

### Training and Fidelity

Therapists in both groups were accredited and received 5 days of refresher training to familiarize them with the therapy manual. F.N. supervised 6 part-time therapists weekly. Three therapists were assigned to each treatment intervention group. Therapists in the intervention group received additional training and supervision in cultural sensitivity. The training was provided separately to prevent cross-contamination between the 2 groups.

### Sample Size Calculation

The primary focus of this study was the feasibility and acceptability of an online CaCBT intervention. Therefore, a sample size was calculated to detect the minimum difference. The sample size was calculated by comparing group changes in the HADS-depression subscale scores. With a 5% significance level and 90% power, we determined that 48 subjects per group will be required for the trial to detect a standardized effect size Cohen's d = 0.68, which is equivalent to a difference in HADS score at the end of the trial of around 2.4 points, assuming a standard deviation of 3.5 found in previous studies from our lab that used the same outcome.^29^ To account for up to 30% attrition, we recruited 140 participants for the study.

### Randomization Procedure

A statistician blind to the recruitment process provided the randomization schedule. The randomization was conducted in blocks of size 2, 4, 6, or 8 and stratified by sex (male, female) and sites (Toronto, Ottawa, Vancouver). Assignments were concealed, meaning that the person assigning subjects to arms was not aware of the assignment until the moment it happened. This was achieved by having individual assignments in sealed, pre-ordered, and numbered envelopes. A statistician created the randomization schedule in R, using a seed so that the process was reproducible. The statistician then prepared the sealed envelopes along with instructions for the randomization.

### Outcomes

Assessments were carried out at baseline, the end of therapy (12 weeks from baseline), and at follow-up (36 weeks from baseline) by raters blinded to group allocation. The primary outcome, feasibility, was measured through engagement, recruitment, and participant retention. Acceptability was measured through feedback from participants and therapists. Participants were also asked to report their satisfaction using the Verona Service Satisfaction Scale.^30^ Working Alliance Inventory (WAI)^31^ was used to measure therapeutic engagement. Secondary outcomes included depression, anxiety, somatic symptoms, and disability. Depression and anxiety were measured using the HADS-depression scale.^32^ HADS is a 14-item self-assessment scale designed to measure anxiety and depression. The maximum score is 21 for depression and 21 for anxiety. A score of 8–10 suggests the presence of borderline cases, while a score of 11–21 indicates abnormal cases. The Bradford Somatic Inventory (BSI) enquires about a wide range of somatic symptoms during the previous month and has 45 items. Scores above 21 indicate depression.^33^ Disability was measured using the World Health Organization's Disability Assessment Schedule 2.0 (WHODAS).^34^ This scale assesses disability due to physical and psychological problems and has been used extensively in various research settings.

### Statistical Analyses

Feasibility and acceptability measures were analysed through frequency and independent sample *t*-tests. For the clinical variable, we followed the CONSORT guidelines for randomized controlled trials.^35^ A statistician blinded to the treatment group conducted the statistical data analysis. Intention-to-treat was used for the primary analysis (subjects were analysed in the group they were randomized to, regardless of compliance with treatment). A mixed-effect model was used for the primary analysis. HADS scores were the dependent variable, and subjects were specified as random effects. The time (baseline, 12 weeks, 36 weeks) by group (CaCBT and standard CBT) interaction was the primary predictor of interest and declared significant if *P* < 0.05. The model also included sex (male, female) and therapists as controls, as these variables were part of the design. Therapists were confounded with the site, which was dropped from the model for this reason. Baseline HADS was also controlled as it is strongly associated with the outcome. Mixed-effect models were estimated through Maximum Likelihood, which accounts for missing values under the Missing At Random (MAR) assumption.^36,37^ A comparison between dropouts and completers on baseline variables was conducted, and variables found to be different at *P* < 0.30 entered a logistic regression model to estimate the propensity of completing the study, which was used to calculate inverse propensity weights for a sensitivity analysis using ANCOVA, aimed at accessing the impact of missingness on the estimate of treatment effect.^38^ A per-protocol analysis with only completers was also conducted to estimate the pure treatment effect. Diagnostic analysis was performed based on residuals and Cook's distance to detect issues related to outliers, influential points, lack of normality, and heteroscedasticity.

## Results

**Figure 1.**
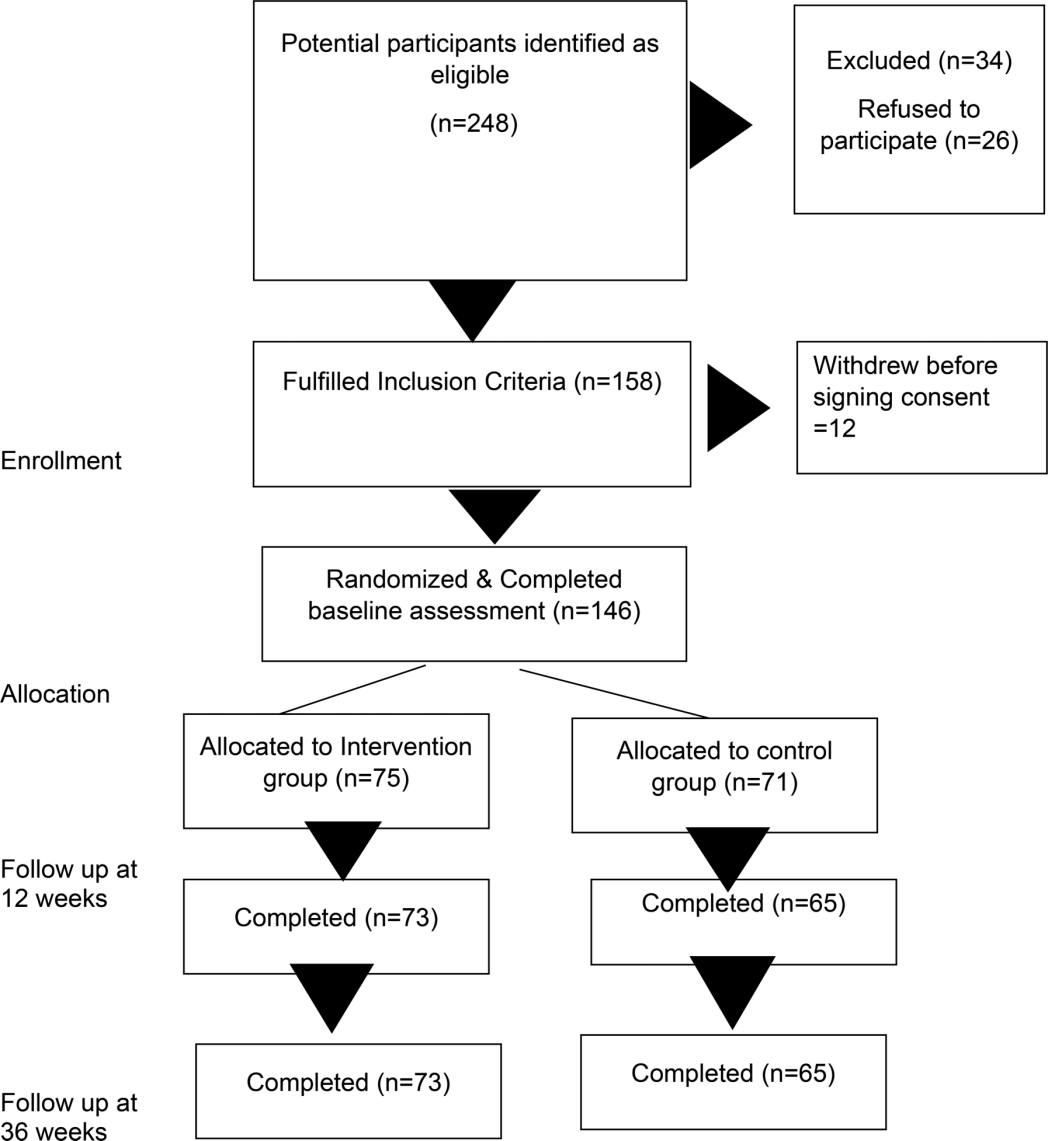
CONSORT flow diagram of the trial.

Response to the online recruitment campaign, which ran between May and December 2021, was excellent. Out of the 146 participants who consented, 75 were randomly assigned to the CaCBT group and 71 to the standard CBT groups. Of these, 57 (39.0%) were from Ottawa, 55 (37.7%) were from the greater Toronto area, and 34 (23.3%) were recruited from Vancouver. Figure 1 summarizes participant flow throughout the study.

The average age of the participant sample was 30.7 (SD = 10) years, of whom 67 (45.9%) were Canadian-born. All the Canadian-born [79 (54.1%)] reported English as their first language. Out of the 79 (54.1%) participants born outside of Canada, 27 (18.5%) were from Pakistan, 26 (17.8%) from India, 8 (5.5%) from Bangladesh, 6 (4.1%) from Sri Lanka, 2 (1.4%) from Nepal, 1 (0.7%) from Afghanistan, and 8 (5.5%) from other south Asian countries. One hundred nineteen (81.5%) of the participants were female, while 26 (18.5%) were male, 1 participant (0.7%) identified as non-binary. One hundred twenty-five (85.6%) were heterosexual, 3 (2.1%) lesbian, 9 (6.3%) bisexual, 4 (2.6%) queer, and 5 (3.4%) other (do not know, prefer no labels, prefer not to answer). Most of the participants were Muslim [*n* = 57 (39.0%). The rest were Hindu [26 (17.8%)], Sikhs [33 (22.6%)], Buddhist [ 6 (4.1%)], Christians [13 (8.9%)], Zoroastrians [2 (1.4%)], Agnostic [12 (8.2%)] Atheist [4 (2.7%)], or other [10 (6.8%)]. Table 1 describes demographic differences between the 2 groups.

**Table 1.** Differences in Demographic Variables and Psychopathology Between the Intervention and Control Groups at the Baseline, Where Figures are (Mean) SD for Age, Years in Canada, and Psychopathology, While the Rest are Number (%).

	Intervention (*n* = 75)	Control (*n* = 71)	P* value
Gender			0.832
Male	13 (17.3%)	14 (19.7%)	
Female	62 (82.7%)	57 (80.3%)	
Age in years	29.9 (9.39)	31.5 (10.7)	0.431
Born in Canada	31 (41.3%)	36 (50.7%)	0.319
Time since arrival in Canada in years^a^	10.5 (12.5)	7.92 (11.4)	0.144
Relationship status			0.734
Single	27 (36.0%)	28 (39.4%)	
In a relationship	48 (64.0%)	43 (60.6%)	
Employment status^b^			
Employment part time	17 (22.7%)	20 (28.2%)	0.454
Employment full time	31 (41.3%)	27 (38.0%)	0.737
Student	20 (26.7%)	19 (26.8%)	1.000
Unemployed	8 (10.7%)	11 (15.5%)	0.464
Annual household income			0.987
<$40,000	21 (28.0%)	19 (26.8%)	
$40,000–$80,000	25 (33.3%)	23 (32.4%)	
>$80,000	25 (33.3%)	25 (35.2%)	
Unknown	4 (5.3%)	4 (5.6%)	
Education^c^			0.529
High school	12 (16.0%)	12 (16.9%)	
College/bachelors	46 (61.3%)	38 (53.5%)	
Master/doctor	16 (21.3%)	21 (29.6%)	
Missing	1 (1.3%)	0 (0%)	
Living situation			0.405
Alone	12 (16.0%)	9 (12.7%)	
Partner (with or without children)	15 (20.0%)	23 (32.4%)	
Other (parent, roommate, children)	48 (64.0%)	39 (54.9%)	
First language English	41 (54.7%)	38 (53.5%)	1.000
HADS-depression subscale score	10.1 (2.83)	10.3 (3.30)	0.479
HADS-anxiety subscale score	14.0 (3.72)	13.9 (3.22)	0.887
HADS total score	24.0 (5.09)	24.1 (4.74)	0.676
BSI total score	29.2 (14.7)	29.0 (13.1)	0.931
WHODAS total score	41.9 (17.5)	42.1 (17.5)	0.836

^a^ If born in Canada, this variable captures the respondent's age.

^b^ Respondents were allowed to select more than 1 employment option. As a result, statistical tests for differences in proportions were conducted for each level.

^c^ Statistical test did not include the missing category.

*P values using the *t*-test for age, number of years in Canada and psychopathology, and chi-square for the rest.

Recruitment was completed within the allocated time. Retention during the trial was excellent. Most of the participants [intervention group = 73 (97.3%), control group = 65 (91.5%)] were available for the end of the intervention assessment (12 weeks from baseline) and for the follow-up assessment (36 weeks from baseline) [intervention = 73 (97.3%), control = 65 (91.5%)]. Most participants in the intervention group 56 (74.7%) attended ≥ 8 sessions, 11 (14.7%) attended 5 to 7 sessions, while for the control group, respective numbers were 50 (66.7%) and 12 (16.0%). Dropouts were low, with 8 (10.7%) participants from the intervention group and 9 (12.0%) from the control group dropping out of therapy in the intervention (<5 sessions).

**Figure 2.**
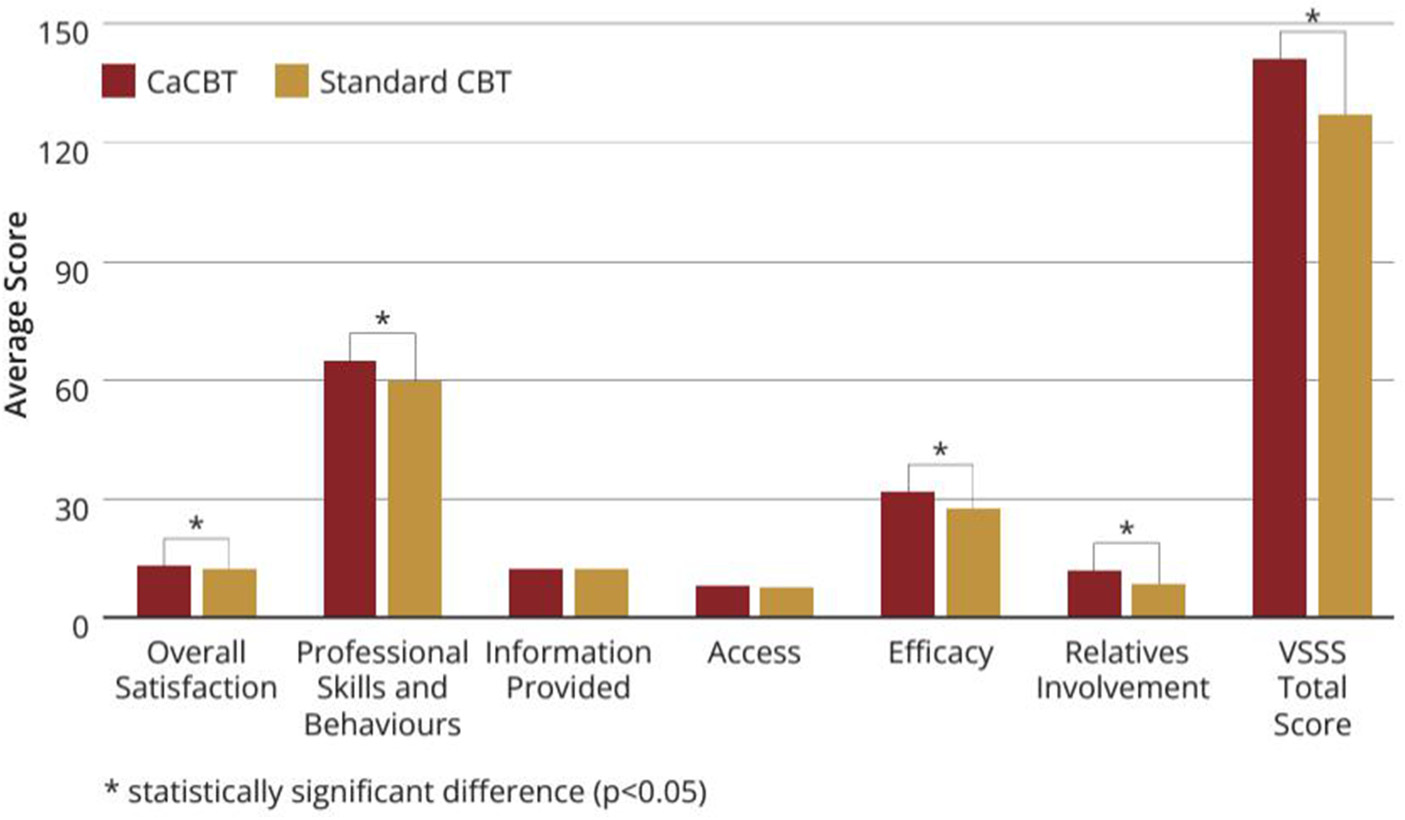
Verona service satisfaction scale.

Participants in the intervention group scored significantly higher on the total Verona Satisfaction Scale [intervention mean, 140.8 (SD = 24.6), control mean, 126.7 (SD = 24.3) (*P* = 0.001)] and overall satisfaction [intervention mean, 13.7 (SD = 2.0), control mean, 12.3 (SD = 2.6) (*P* = 0.025)]. In other areas, professional skills and behaviours [intervention mean, 65.1 (SD = 7.7), control mean, 59.4 (SD = 9.8) (*P* = 0.025)], efficacy [intervention mean, 31.5 (SD = 5.5), control mean, 27.3 (SD = 5.3) (*P* < 0.001)], relative's engagement [intervention mean, 11.6 (SD = 9.7), control mean, 27.3 (SD = 5.4) (*P* = 0.035)], but not on info provided [intervention mean, 12.21 (SD = 2.3), control mean, 12.0 (SD = 2.2) (*P* = 0.689)], and access [intervention mean, 7.7 (SD = 2.3), control mean, 7.5 (SD = 2.3) (*P* = 0.659)] (see Figure 2). The participants also scored higher on both the client [intervention mean, 219.7 (SD = 25.7), control mean, 208.2 (SD = 35,0) (*P* = 0.036)] and the therapist [intervention mean, 227.3 (SD = 18.7), control mean, 202.4 (SD = 27.4) (*P* < 0.001)] subscales of the WAI in the intervention group.

Analysis of secondary clinical outcomes (measured via the HADS, BSI, and WHODAS 2.0) found a smaller than expected treatment effect size (difference of 0.6 points in HADS at 6 months, Cohen's d = 0.26 using between-subject standard deviation) but a very large time effect (change of 8.1 (SE = 0.5) points in HADS from baseline to 6 months, Cohen's d = 2.0 using within-subject standard deviation), which was similar in both groups. The mean scores of participants on depression subscale were below caseness in both the intervention and control groups (see Table 2).

**Table 2.** Differences Between the Treatment and Control Groups Over Time, Controlled for Initial Differences.

	Treatment (*n* = 75) (mean) SD	Control (*n* = 71) (mean) SD	Mean difference (CI) (controlled for baseline)	*P* *
HADS depression				
Baseline	10.12 (2.83)	10.3 (3.30)	0.20 (−0.80, 1.20)	0.479
12 weeks	6.43 (3.28)	5.74 (3.63)	0.73 (−0.47, 1.94)	0.233
36 weeks	5.85 (4.10)	6.22 (3.58)	0.41 (0.67, −0.91)	0.534
HADS anxiety				
Baseline	14.0 (3.72	13.9 (3.22)	−0.11 (−1.24, 1.02)	0.887
12 weeks	9.6 (3.90)	9.5 (3.23)	−0.16 (−1.40, 1.06)	0.787
36 weeks	9.7 (3.96)	9.8 (3.52)	0.08 (0.65, −1.22)	0.901
HADS total				
Baseline	24.0 (5.09)	24.1 (4.74)	0.56 (−1.47, 2.60)	0.676
12 weeks	15.3 (6.53)	15.9 (5.21)	0.66 (−1.57, 2.60)	0.585
36 weeks	15.5 (7.17)	16.0 (6.17)	0.50 (−1.82, 2.82)	0.688
BSI				
Baseline	29.2 (14.7)	29.0 (13.1)	−0.19 (−4.71, 4.36)	0.931
12 weeks	19.5 (11.96)	21.6 (11.90)	2.1 (−2.0, 6.2)	0.321
36 weeks	21.2 (15.27)	20.6 (13.0)	2.0 (−2.0, 6.2)	0.794
WHODAS02				
Baseline	41.9 (17.5)	42.1 (17.5)	0.18 (−5.7, 6.1)	0.836
12 weeks	29.2 (17.6)	31.6 (17.4)	2.3 (−4.0, 8.6)	0.476
36 weeks	29.6 (20.0)	29.0 (17.1)	−0.62 (−7.45, 6.21)	0.875

*P-values using multiple regression.

## Discussion

This is the first study of online CaCBT for depression and anxiety among South Asian populations in North America and Western Europe. The results of this feasibility study show that CaCBT was an acceptable and feasible intervention among Canadian South Asians. While not a definitive trial, participants engaged with CaCBT reported a high satisfaction score and provided positive feedback on the intervention. While the focus of this study was not treatment outcomes, it confirms existing evidence that suggests that CaCBT is effective in treating symptoms of depression and anxiety. However, most research has compared CaCBT with a waitlist or a control group that received Treatment As Usual.^39^ We must also emphasize that comparing 2 effective interventions offered by qualified therapists seldom see any differences in comparisons.^40^

Culturally adapted CBT has been developed and tested for the local population in South Asia. More than 20 RCTs have been reported to have shown CaCBT to be effective.^15^ The current study adds to the literature on culturally adapted CBT interventions for this population. Due to the COVID-19 pandemic, the study was conducted online. During the recruitment stage, we found that social media (Twitter, Facebook, and Instagram) were most helpful in increasing our recruitment numbers. Using technology to communicate, engage, and maintain contact for follow-up was immensely useful, and participants responded quickly. Many participants shared that virtual therapy had increased their accessibility. This could have been because the appointments were easy to arrange, with no cost or time required to travel and no need for childcare or taking time off work. It is also possible that these participants felt less stigmatized attending therapy from the comfort of their homes. Almost all participants were self-referred, which may indicate a higher motivation in the SA Canadian community to participate. However, these participants might not be typical SA Canadians.

Culturally adapted CBT has been tested in digital formats, using online and mobile phone applications and found to be effective^41,42^ as self-help or guided self-help. However, as far as we are aware, culturally adapted CBT has not been tested using online therapy. This is also the first trial of culturally adapted CBT delivered through a dedicated online communication platform. Online therapy delivery also led to difficulties connecting and following up with participants and technological challenges. However, overall, the retention and recruitment rates were excellent.

On the other hand, some participants felt that having therapy online was a barrier to being fully open and transparent with their therapist. Other participants found it difficult to find private space in their homes for the sessions. Some participants felt disconnected during online therapy sessions, as if they were not really “in” the therapy sessions because they were virtual.

Despite interpreters being available for various SA languages, potential participants were not interested in attending sessions with an interpreter present if the therapist did not speak their language. Their reluctance was likely due to feelings of stigma and shame about mental illness, which could have been exacerbated by having a third person (the interpreter) present in the session. Having interpreters present could also raise participants’ concerns about confidentiality, trust, and engagement, which are so crucial to the therapeutic process. Participants in this group were young, highly educated people.

The study's findings suggest that SA communities are open to accepting input from formal services that meet their needs based on their values, beliefs, family dynamics and language preferences. Policy decision-makers and programme managers need to consider the mental health needs of SA communities when developing and evaluating mental health services across the country and when implementing and using the training packages with South Asians in their communities. Both Ontario and Quebec have launched structured psychotherapy programmes. However, there are no plans to address issues of equity for immigrant, refugee, ethnocultural, or racialized (IRER) populations. The findings from this study provide initial evidence for the Canadian population. However, these results must be confirmed in fully powered trials focusing on cost-benefit analysis.

We also report higher rates of satisfaction on all subscales of Verona service satisfaction scores and working alliance among therapists in the intervention group. There is evidence that higher satisfaction scores are associated with increased completion rates and superior clinical outcomes^43^ and low rates of burnout among therapists.^44^ However, this assumption has been challenged.^45^ Similarly, it has been suggested that working alliances and therapy outcomes have a strong association. The current study failed to confirm this finding.

### Limitations

There are multiple reasons why clinical outcomes did not reach statistical significance in favour of intervention. The depression scores in both groups were low at the baseline [intervention, HADS-depression subscale mean = 10.1 (SD = 2.83) and control, HADS-depression subscale mean = 10.3 (SD = 3.30)], indicating there was not much room for improvement. Participants in both groups self-referred and were highly motivated. They were also recruited from online platforms, indicating high comfort with the online intervention. Finally, they were young and educated. We also did not assess participants’ acculturation level due to resource issues. This is especially important since the number of years in the host culture will likely influence the acculturation level. Another limitation of this study was the over-representation of women in our study. Most of whom were Muslims. Muslim women likely felt the option of online therapy from the comfort of their homes was less stigmatizing. Therapists in both groups were supervised by F.N., which might have introduced a source of bias. It is also likely that the culturally adapted CBT requires further modification to be delivered online. In addition, it would be interesting to know the relative efficacy of regular CBT delivered by culturally matched therapist-patient dyads compared to culturally adapted CBT delivered by therapists of different cultures than their patients.

There is a need to explore this area further and test culturally adapted CBT in a fully powered RCT with a clinical population that can also compare online and in-person therapy.

## Conclusions

This is the first study of an online, culturally adapted intervention for Canadian South Asians. The intervention was feasible and acceptable, with excellent recruitment and retention rates. Participants in both groups showed improvement, meaning that culturally adapted CBT is as effective as standard CBT in reducing symptoms of depression and anxiety. However, the intervention arm participants reported higher satisfaction and therapeutic engagement rates. This study raises some important questions and issues that need to be addressed to advance the field of culturally adapted psychological interventions. There is a need to replicate this work with improved methodology in a fully powered RCT.
